# Fatal heat stroke based on foudroyant irreversible multiple organ dysfunction in German summer

**DOI:** 10.1515/iss-2023-0013

**Published:** 2023-09-19

**Authors:** Eric Lorenz, Joerg Herold, Uwe Lodes, Frank Meyer

**Affiliations:** Department of General, Abdominal, Vascular and Transplant Surgery, University Hospital, Magdeburg, Germany; Department of Angiology, Municipal Hospital of Darmstadt, Darmstadt, Germany; Department of Anaesthesiology and Intensive Care, Municipal Hospital (AMEOS-Klinikum), Schönebeck, Germany

**Keywords:** heat stroke, multiple organ failure, disseminated intravascular coagulation, cooling, intensive care, liver transplantation

## Abstract

**Objectives:**

Heat stroke is a serious condition that might lead from moderate organ impairment to multiple organ dysfunction syndrome. Appropriate diagnosis-finding, fast initiation of cooling and intensive care are key measures of the initial treatment. Scientific case report based on i) clinical experiences obtained in the clinical management of a particularly rare case and ii) selected references from the medical scientific literature.

**Case presentation:**

We present a case of a young and healthy construction worker who suffered from an exertional heat stroke with a body core temperature exceeding 42 °C by previous several hour work at 35 °C ambient temperature. Heat stroke was associated with foudroyant, not reversible multiple organ dysfunction syndrome, in particular, early disturbed coagulation, microcirculatory, liver and respiratory failure, and subsequent fatal outcome despite immediate diagnosis-finding, rapid external cooling and expanded intensive care management.

**Conclusions:**

Basic knowledge on an adequate diagnosis(-finding in time) and treatment of heat stroke is important for (almost each) physician in the summertime as well as is essential for the initiation of an appropriate management. Associated high morbidity and mortality rates indicate the need for implementation of standard operation protocols.

## Introduction

Heat stroke is a life-threatening syndrome secondary to failure of the thermo-regulation system caused by hyperthermia with a body core temperature of more than 40 °C. Consequently, dysregulation or failure of multiple organs can be observed. Clinical symptoms range

**Table:** 

from	– delirium
	– seizures,
	– coma,
	– rhabdomyolysis,
	– shock with consecutive electrolyte and acid-base abnormalities
to	– acute renal and liver failure (as well as)
	– acute respiratory distress syndrome (ARDS) and
	– disseminated intravasal coagulation (DIC).

Mortality rates up to 50 % are reported [1]. Etiologically, heat stroke can be categorized in two forms:

– Exertional heat stroke (EHS) is caused by strenuous muscular exercise and occurs mainly in younger active persons – in contrast,
– Classical heat stroke (CHS) is caused by environmental heat and occurs primarily in elderly persons [2].

The two variants may or may not be accompanied by each other. However, EHS occurs especially in

– athletes (and)
– occupational workers

when exaggerated acute phase response and altered heat shock response might lead from compensated heat stress to decompensated heat shock with severe complication(s) [1].

In addition, as you may derive from Tables 1 and 2, there seems to be a male predominance and an age range from 25–44 years of age [3], [4], [5], [6], [7], [8], [9].

**Table 1:** Case series of liver transplantation following heat shock-induced acute liver failure (search engine, Pubmed^®^; Search query, “heat stroke” and “liver transplantation”; time frame, 2018–2023 – chronological order).

Author	Year of publication	Journal	Gender	Age [yr.]	Patients activities
Lin et al. [1]	2022	*ACG Case Rep J*	Male	44	Marathon running
Martins et al. [2]	2022	*Exp Clin Transplant*	Male	28	Running
Bi et al. [3]	2020	*Hepatology*	Males (n=2)		Running, exercising
Ichai et al. [4]	2019	*J Hepatol*	Males (n=24)	25–37	Running, marching
Martinez-Insfran et al. [5]	2019	*Transplant Proc*	Male	32	Working outside
Figiel et al. [6]	2019	*World J Clin Cases*	Males (n=4) Female (n=1)	24–39	Running
LaMattina et al. [7]	2018	*Transplant Proc*	Male (n=1)		Exercising

The *aim* of this scientific case report was – based on current data, reports and references from the present scientific literature and own clinical experiences – to describe an extraordinary young patient with exertional heat shock from daily clinical practice including

i) the causes and consequences of his disfavorable course (caused by a not reversible multiple organ dysfunction syndrome [MODS], which was mainly characterized by disseminated intravasal coagulation due to liver failure) as well as
ii) the details of an appropriate diagnostic management and a step-wise therapeutic approach up to aspects of organ replacement as a possible ultimate treatment option.

### Case report

A 31-year old construction worker was working outside on July 3rd, 2016. At the start of his shift at 8:00 a.m., the ambient temperature was 24 °C and rose up to 35.6 °C at 2:00 p.m. The wind speed was approximately 7 km/h all morning. The humidity was 44 %. In the afternoon, the man complained about dizziness. Suddenly, he collapsed while walking and lost consciousness. Upon arrival of the paramedics, the patient was comatose with a Glasgow Coma Scale of 3 (GCS – E1V1M1) and was gasping as a sign of respiratory insufficiency counting for a massively reduced circulation/hemodynamic arrest. The peripheral oxygen saturation was 30 % underlining the assessment, the blood pressure was 100/60 mmHg and the patient suffered from a narrow QRS-complex tachycardia with a frequency of 180 bpm with no precise information how long the status had already persisted. The patient was intubated and paramedics tried to slow down heart rate by using Amiodaron (300 mg) and beta-blockers. Initially, the assessment of body core temperature was impossible due to exceeding of the temperature scale but throughout outpatient clinic service, the body core temperature was measured at 42.3 °C. According to the patient’s brother, the medical history was unremarkable. The patient’s brother denied usage of drugs, intake of medications or any family history of heart problems. The patient was then admitted to the nearest emergency room with suspected diagnosis of heat stroke, pulmonary embolism or acute heart attack. Upon arrival, tachycardia was found. Blood chemistry test showed pathologically elevated levels of myocardial markers such as

– creatine kinase-MB (2.03 μmol/s.L) and
– troponin-T (0.319 ng/mL), myoglobin (18,947 μg/L) as well as

**Table:** 

elevated	– aspartate aminotransferase	(9.24 μmol/s.L),
	– alanine aminotransferase	(3.01 μmol/s.L),
	– glutamate dehydrogenase	(1,500 nmol/s.L.),
	– creatinine	(270 μmol/L),
	– procalcitonin	(6.3 ng/mL),
	– D-dimer	(2.6 mg/L) and
	– spontaneous partial thromboplastin time	(164.7 s).

Blood gas analysis showed a mild lactic acidosis (4 mmol/L) and high potassium (5.6 mmol/L). The remaining electrolytes and blood sugar were within normal range. To rule out ischemic heart disease, coronary angiography was performed without any sign of impaired ejection fraction, coronary atherosclerosis or cardiac hypokinesia.

Cranial, thoracic and abdominal computed tomography (CT) was unremarkable except for fluid-filled small bowel and colon with signs of wall-thickening of the small bowel so that the clinical diagnosis suspected a massive gastrointestinal infection amongst other reasons. Supportive therapies such as

– intravenous fluids were given,
– compensation of coagulation was initiated (and)
– antibiotics were administered.

**Figure 1:**
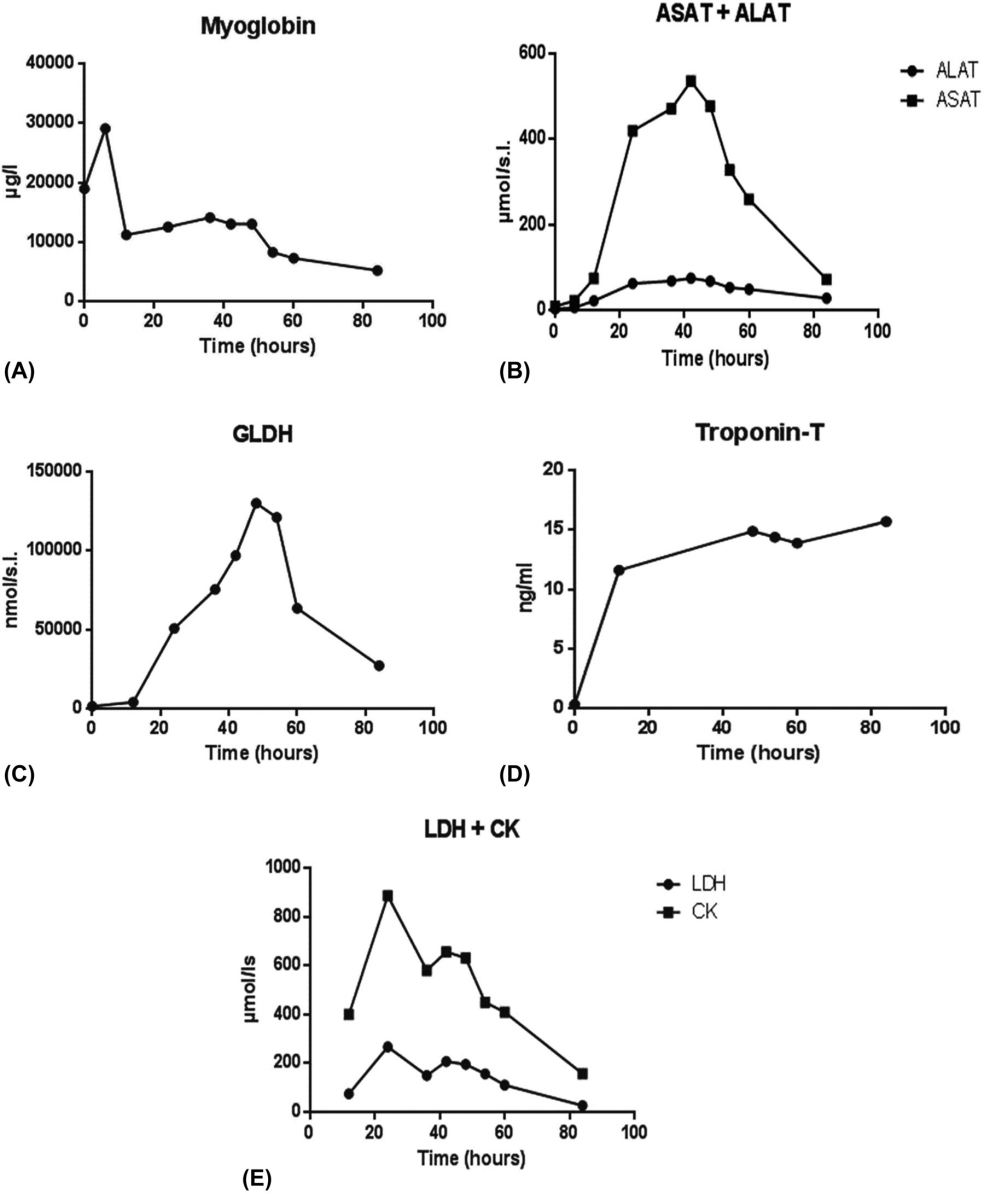
Time course of laboratory parameters since patient’s admission. (A) Myoglobin, (B) AST and ALT, (C) GLDH, (D) troponin T, (E) LDH and CK.

External cooling was started. Six hours after starting external cooling, the body core temperature was within normal range. The patient suffered from an acute kidney injury (AKI) at least partially related to a crush-syndrome caused by elevated myoglobin. Therefore, the patient was admitted to the intensive care unit and treated with continuous veno-venous hemofiltration (CVVH) with a myoglobin filter. In the course of events, myoglobin-level declined as a result of performing a myoglobin filter (Figure 1A) but the leading clinical findings were progressive microcirculatory failure as well as liver and respiratory failure – liver transplantation was discussed as ultima ratio. In the course of events, there was a continuous and rapid increase of liver enzymes such as AST, ALT and GLDH with a maximum at 42 and 48 h after admission to the hospital, respectively (Figure 1B and C). Due to continuous increases of Trop-T, CK and LDH (Figure 1D and E), repeated echocardiography was performed showing moderate regional wall motion abnormalities but without impairment of left ventricular pumping function.

**Figure 2:**
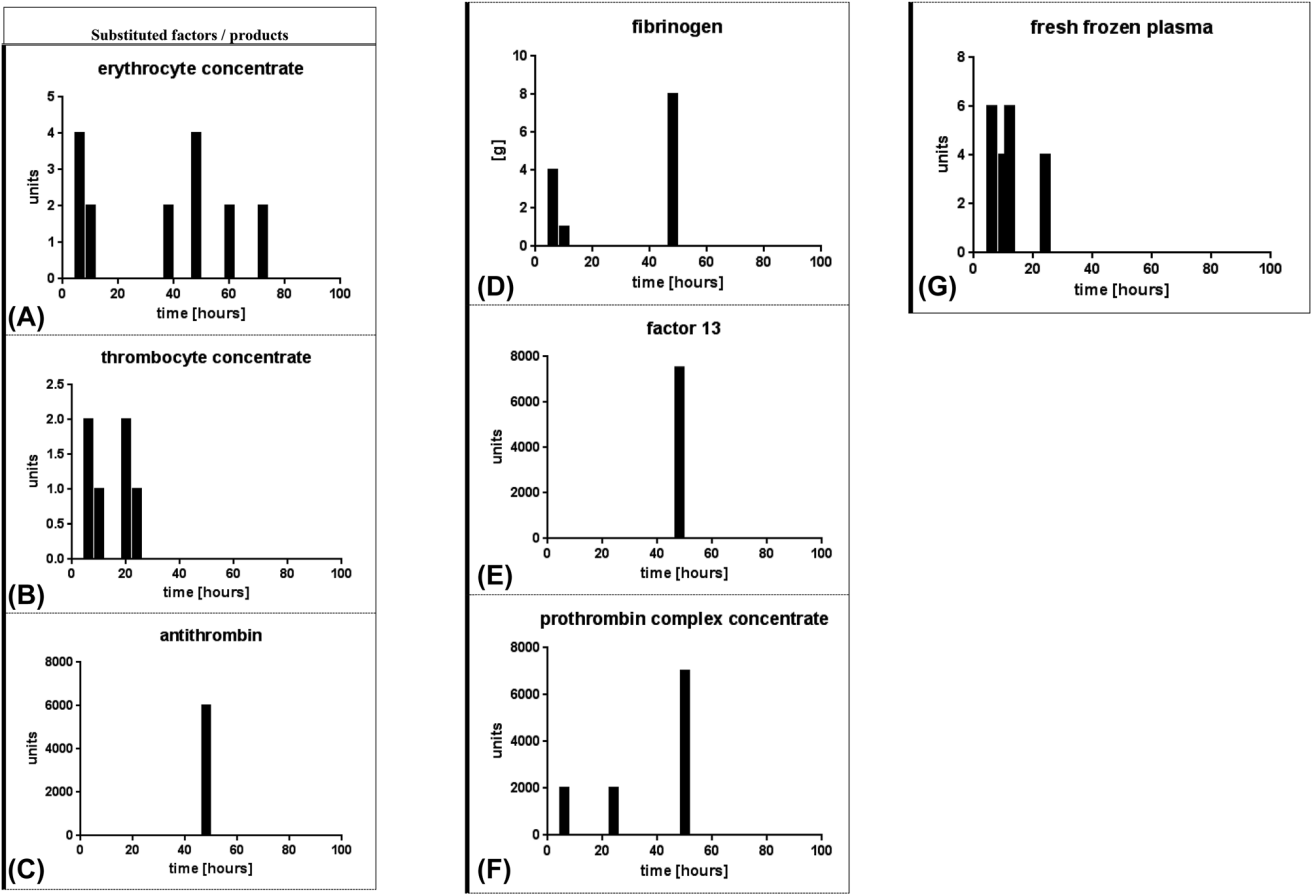
Substitution of blood products and factors according to the course of various aggregation- and coagulation-relevant parameters and factors. (A) Red cell packs, (B) platelet concentrate, (C) antithrombin, (D) fibrinogen, (E) factor 13, (F) PPSB, and (G) FFP’s.

Substitution of blood products and factors according to the course of various aggregation- and coagulation-relevant parameters and factors (Figure 2)

As depicted in detail in Figure 2A–G, there was a persisting, variable and recurrent need for blood products and aggregation as well as coagulation factors during the heat stroke course – ongoing and massive substitution measures, such as transfusion of red cell packs (panel 2.A) at least at the beginning (platelets – 2.B; FFP’s – panel 2.G) or once in combination in the middle of the course (antithrombin – panel 2.C; fibrinogen – panel 2.D; factor 13 – panel 2.E; and PPSB – panel 2.F).

Due to a rapid worsening of various organ functions as part of the MODS, the patient could not be seriously considered for a liver transplant. Approximately 72 h after hospital admission, the patient showed dilation of pupils without light responsiveness prompting to immediate cranial CT scan, which revealed an advanced brain edema with herniation of the brainstem as well as hypoxic areas. Due to missing brainstem reflexes and unfavorable prognosis, there was no neurosurgical intervention. The patient died of advanced and severe MODS 4.5 days after admission to the hospital.

## Discussion

Heat stroke is a severe emergency that can lead to the patient’s death if not treated properly by immediate reduction of body core temperature [1]. Mortality rates up to 62 % with a median survival time of 13 days have been reported [10]. Heat stroke can be accompanied by multiple organ dysfunction syndrome in 75 % of cases [11]. Environmental conditions such as ambient temperature and humidity play an important role in the emergence of a heat stroke but abnormal endogenous thermogenesis and/or heat-loosing mechanisms seem to be as well of etiological relevance [12]. In particular, Rae et al. assessed that the hyperthermic states experienced by their cases presented may have resulted from failure of their heat-losing mechanisms. Alternatively, they might have resulted from excessive endothermy, triggered by physical exertion and other unknown initiating factors. Excessive endothermy should be considered in cases of heatstroke that occur in mild to moderate environmental conditions [12].

Here, a case of a construction worker suffering from an exertional heat stroke (EHS) with a body core temperature exceeding 42 °C is presented that led to a MODS and resulted in the patient’s death [1]. It remains speculative whether MODS/consecutive liver failure are a result of ischemia – in addition, hemodynamic instability because of tachycardia (or even ventricular fibrillation) and ischemia, which lead to MODS (as a theory), is possible. Perhaps, the fast rhythm is caused by a hyperthermia-induced Brugada syndrome (ion channel disease with electrical disturbance of heart function without detectable alteration of the heart tissue [structure], in which life-threatening cardiac irregularities can occur).

In general, heat dissipation can be improved by cooling methods using

– conduction (temperature gradiant),
– evaporation (water vapor pressure) and
– convection (velocity of air over the skin) [13].

Regarding conduction placing the patient in a tub with iced water while massaging the extremities for vasodilatation is the most frequently used technique with relatively low mortality rates [13]. Besides that, application of ice packs seems to be reasonable. However, here a mortality rate of 22 % was reported [13]. Alternative methods such as endovascular cooling or lavage of colon, stomach or bladder with cold water might be successful in reducing body temperature [13], [14], [15]. Although, there are few reports on exertional heat strokes – evaporation by using fans especially in combination with wet gauzes or spraying of atomized water onto the skin seems to be very effective [13]. In general, usage of iced water is very effective especially while keeping the skin vessels dilated by massage. However, despite immediate admission of fluids upon arrival of paramedics, external cooling when admitted to the emergency room and support of organ functions on the intensive care unit might be insufficient to reduce body core temperature in some cases [12]. It is suspected that a prolonged temperature reduction time might be caused by excessive endogenous thermogenesis that may lead to fatal outcome. Despite external cooling in the presented case, it took about 6 h to reach regular body core temperature. In addition, the patient showed elevated levels of procalcitonin (6.3 ng/mL) without any sign or proof of concomitant infection. These findings coincide with other publications [16]. However, antibiotic therapy seems to be reasonable. In the course of events, the presented patient died from consequences of MODS, with significantly elevated tissue enzymes caused by direct thermal damage and impaired macro- and microhemodynamics. Compared to other studies with low mortality rates [13], it took longer to reduce body core temperature. It remains unclear whether excessive endogenous thermogenesis was at least partially responsible for the prolonged cooling period.

In particular, it was challenging to clarify primary differential diagnosis (each indicated by various aspects) with regard to

– *heat stroke* (massively elevated body core temperature >42 °C, subsequently disturbed coagulation [prolonged prothrombin time; requiring compensation], microcirculatory, liver and respiratory failure),
– *pulmonary embolism* (elevated D-dimers, subsequently respiratory failure) or
– *acute heart attack* (elevated laboratory parameters such as heart enzymes and rhythmological alterations indicated in electrocardiogram).

In this context, there were factors that indicated an unfavorable outcome in the early stage of the heat shock [10]. Accordingly, the patient showed

– an initial GCS of 3,
– a body core temperature of more than 42 °C,
– prolonged prothrombin time (due to liver failure; requiring substitution of coagulation factors, e.g., by administration of fresh frozen plasma) and
– immediate need for vasoactive drugs

as early sign of a worse prognosis. The patient was considered for high-urgency liver transplantation due to acute und rapidly progressive liver failure. Interestingly, there are rare data that indicate a long-term survival (longer than 1 year) after liver transplantation due to an acute hepatic failure following exertional heat stroke [17]. However, the patient died prior to a possible transplantation due to rapid worsening of his clinical status.

## Conclusions

Heat stroke with consecutive treatment is of importance for physicians especially in the summertime. Rapid diagnosis of heat stroke and immediate cooling as well as additional intensive care measures are key factors to preserve organ function, again, in particular, coagulation, microcirculation, lung and liver. Since exertional heat stroke with MODS can be resistant to external cooling, standard operation procedures should be adjusted using alternative or additional cooling methods. High survival rates can be achieved by using methods that facilitate or maintain vasodilatation of skin vessels for improved conduction.

Although, there are not many cases published in the literature regarding liver transplantation following heat-induced liver failure, it should be seriously considered as salvage therapy if appropriate.

## Supplementary Material

Supplementary Material
