# Supplementary material for: Fatal heat stroke based on foudroyant irreversible multiple organ dysfunction in German summer
Source: Innov Surg Sci. 2023 Sep 19;8(2):129–34. doi: 10.1515/iss-2023-0013 (PMC10696933; doi:10.1515/iss-2023-0013)
Supplement: Supplementary file 1 — Supplementary Material [file j_iss-2023-0013_suppl_001.pdf]

Reviewer Assessment

E. Lorenz et al.: Fatal heat stroke based on foudroyant irreversible multiple organ dysfunction syndrome in German summer

Reviewers' Comments to Original Submission

Reviewer 1: Paasch, Christoph

Date received: 03-Apr-2023
Reviewer recommendation: **Return to author for major modifications**
Reviewer overall scoring: **High**

Comments to author:
The authors presented an interesting and well-written case of a fatal heat stroke. In general, the manuscript should be published from my perspective. It is a rare disease and medical stuff should be aware. A Revision is needed.
The manuscript is too long and should be shortened.

Title: The title should be shortened.

Abstract: With > 270 words the abstract is too long from my perspective

Introduction: The authors should provide some information on incidence and gender distribution.
Page 4, Lane 41-43 The authors should not tease the report of the case.

Case report:
Page 5, Lane 9: Substance name should be used instead of „Cordarex"
Page 5, Lane 25-38 Abbreviations not needed and should be deleted.
Page 5 Two times furthermore should be avoided.
Page 6 Several times therefore should be avoided.

Discussion: The discussion section is lacking detailed information on comparable cases. A summarized table listing similar case reports would be helpful.
The title suggests a deep discussion on "is liver transplantation a reasonable ultimate treatment option?" Here the authors should provide a discussion!

Page 6, Lane 57 and
Page 5, Lane3 " abnormal endogenous…." It would be interesting to get more detailed information on these hypothesis!
Page 8, Lane 22 Please, rephrase the first sentence of the limitation section for better understanding.

Figures/Tables: To make the manuscript shorter and shapelier, ONE summary and colour illustration should be made.
Figure 3 is not needed from my perspective; the authors should roughly mention the amount in the text using brackets…

Conclusion: Far too long. There should be short take home message!

Reviewer 2: Hansen, Michael

Date received: 30-May-2023
Reviewer recommendation: **Return to author for major modifications**
Reviewer overall scoring: **Medium**

Comments to author:
Dear colleagues, thank you for reporting this interesting case. In my clinical career I´ve seen 2 cases like this one. I read the paper as a physician of intensive care and invasive cardiology. The time for this review was short, please excuse the short form.

I have some recommendations:

1. hypothermia should be a keyword

2. Abstract, line 18 conclusion, in my mind it is better in this form: diagnosis and treatment of heat stroke is important for physicians in the summertime.

3. Introduction, line 12, better: heat stroke can be categorized in two forms.

4. Case report, page 4, line 3: the patient was gasping as a sign of respiratory insufficiency

5. In case of collapsing and GCS 3 with gasping it´s a high possibility of massive reduced circulation/hemodynamic arrest. The first measured saturation of 30% is underlining the theory. How long was the time without sufficient circulation? There was no help from bystander?

6. Case report :"Initially, the assessment of body core temperature was impossible due to exceeding of the temperature scale but throughout outpatient clinic service, the body core temperature was measured at 42.3°C." What does it mean? Core temperature was initially higher and because of later measurement the true value is missed?

7. ECG: cardial axis is unclear, sinus rhythm, we have not! a typical right bundle branch block. It seems to be a brugada type 2 with st-elevation in v1/v2 and typical saddleback morphe. On the ACC 2022 a poster was presented of a hypothermia induced brugada. Maybe was the first rhythm a VF with a spontaneous conversion into sinus rhythm and the liver failure/MODS are a result of ischemia?

8. In my mind, the theory of hemodynamic instability because of tachycardia/ventricular fibrillation and ischemia, which leads to MODS is possible. Maybe the fast rhythm is caused by a hyperthermia induced Brugada-Syndrome. Do you have any information about ecg-dynamics?

9. Page 5 , line 3: "In the course of events, myoglobin-level was declining (Fig. 2A) but the leading clinical findings were progressive microcirculatory failure as well as liver and respiratory failure"
Maybe better: Myoglobin-level declined as a result of performing a Myoglobin filter. The clinical situation was worsening with progressive .....

10. Page 7 line 13: A limitation of this scientific report is the only case based on which the problem of heat stroke is described.
better: The meaningfulness of this report based on a single case is limited.

11. The discussion of liver failure and liver transplantation as an ultimate treatment option is very rare. Under the search terms ((heat stroke) AND (liver failure)) AND (liver transplantation) are nearly 40 results, the latest is from 2022.

12. The references are not current

I hope, my recommendations are helpful for presenting this case
I wish a successful process.

Authors' Response to Reviewer Comments

Date received: 26-Jun-2023

Response to Reviewer 1:

I.) The reviewer's 1st statement was: A Revision is needed.

RESPONSE: As you can see, the manuscript has been substantially revised using "Word"-based marking of changes, in particular, in response to each single comment and critic by the reviewers - see also there.

II.) The reviewer commented: The manuscript is too long and should be shortened

RESPONSE: The manuscript has been substantially shortened as demanded by the reviewer - done!

III.) The reviewer criticized: Title: The title should be shortened.

RESPONSE: The title has been shortened as suggested by the reviewer - done!

IV.) The next critic was: Abstract: With > 270 words the abstract is too long from my perspective

RESPONSE: The “Abstract“ section has been substantially shortened too as requested by the reviewer - done!

V.) The reviewer’s next comment was:

Introduction: The authors should provide some information on incidence and gender distribution.

Page 4, Lane 41-43 The authors should not tease the report of the case. Done!

RESPONSE: As you may derive from Table 1, there seems to be a male predominance and an age range from 25 - 44 years of age. This contents were added to the text (“Introduction“ section) as the reviewer asked for - as follows:

“ ... with severe complication(s) (1).

In addition, as you may derive from Table 1, there seems to be a male predominance and an age range from 25 - 44 years of age (3-9).

The aim of this scientific case report was ... “

VI.) Next, the following details were raised:

Case report:

Page 5, Lane 9: Substance name should be used instead of „Cordarex“ Done!

Page 5, Lane 25-38 Abbreviations not needed and should be deleted Done!

Page 5 Two times furthermore should be avoided Done!

Page 6 Several times therefore should be avoided Done!

RESPONSE: All the single suggestions were taken into account - see also the manuscript text.

However, usually if abbreviations are used, they need to be explained if used for the first time within the text - in preparation of using them further below, the authors thought it is a good idea to introduce the abbreviations at this position of the text.

VII.) In addition to a critic, a suggestion was made by the reviewer: Discussion: The discussion section is lacking of detailed information on comparable cases. A summarized table listing similar case reports would be helpful.

RESPONSE: Please, see RESPONSE to point “V.)“ - there is a table (Table 1) “listing similar case reports“.

VIII.) The following additions were demanded by the reviewer: The title suggest a deep discussion on “is liver transplantation a reasonable ultimate treatment option?” Here the authors should provide a discussion!

RESPONSE: The authors tried to follow as good as possoble the reviewer’s orientation (as marked in the revised text) - however, this needed to be balanced by the additiopnal reviewer’s demand to shorten the whole manuscipt.

IX.) In further detail, the reviewer asked: Page 6, Lane 57 and Page 5, Lane3

“ abnormal endogenous….” It would be interesting to get more detailed informations on these hypothesis!

RESPONSE: To report in more detail, the following phrase was added to the text following the reviewer’s instruction appropriately:

“ … be as well of etiological relevance (5). In particular, Rae et al. assessed that the hyperthermic states experienced by their cases presented may have resulted from failure of their heat-losing mechanisms. Alternatively, they might have resulted from excessive endothermy, triggered by physical exertion and other unknown initiating factors. Excessive endothermy should be considered in cases of heatstroke that occur in mild to moderate environmental conditions (5).

Here, a case of a construction worker … ”

X.) The reviewer demanded: Page 8, Lane 22 Please, rephrase the first sentence of the limitation section for better understanding.

RESPONSE: Based on the serious demand by the reviewers to shorten the whole manuscript, the “Limitations“ aspect was completely erased.

XI.) The reviewer suggested: Figures/Tables: To make the manuscript shorter and shapelier, ONE summary and color illustration should be made.

RESPONSE: To balance the two reviewer’s suggestions, comments and critics appropriately (demanded additions versus demand for shortening single sections and the manuscript in total), we pursued the compromise to only shorten substantially the summary/“Conclusion“ section - many thanks for your understanding in advance!

XII.) The reviewer mentioned: Figure 3 is not needed from my perspective, the authors should roughly mention the amount in the text using brackets…

RESPONSE: Again, as mentioned above in the RESPONSE to reviewer’s point “XI.“, to balance the two reviewer’s suggestions, comments and critics appropriately (demanded additions versus demand for shortening single sections and the manuscript in total), we pursued the compromise to keep the figure but, on the other hand, to avoid extending the text - many thanks for your understanding in advance!

XIII.) Final critic by the reviewer was:

Conclusion: Far too long. There should be short take home message!

RESPONSE: According to the reviewer’s demand, the “Conclusion“ section was substantially shortened.

Response to Reviewer 2:

1. The reviewer suggested: hypothermia should be a keyword

RESPONSE: Many thanks - yes, “Hypothermia“ was added tot he keyword list as suggested.

2. The reviewer stated: Abstract, line 18 conclusion, in my mind it is better in this form: diagnosis and treatment of heat stroke is important for physicians in the summertime.

RESPONSE: The reviewer’s suggestion was followed - Done!

The phrase is now as follows:

“ … intensive care management.

Conclusion:

Basic knowledge on an adequate ddiagnosis(-finding in time) and treatment of heat stroke is important for (almost each) physicians in the summertime asis well as is essential for the initiation of an appropriate management.

Heat stroke with compelling consecutive treatment is of importance for physicians especially in the summertime. Associated high morbidity and mortality … ”

3. The next reviewer’s suggestion was: Introduction, line 12, better: heat stroke can be categorized in two forms.

RESPONSE: Thanks fort he better English expression - as follows:

“ … are reported (1). Etiologically, heat stroke can be categorized in two forms:

- Exertional heat stroke (EHS) is caused by strenuous muscular exercise and occurs mainly in

younger active persons - in contrast,

- Classical heat stroke (CHS) is caused by environmental heat and occurs primarily in elderly persons (2).

The two variants may or … “

4. The reviewer commented:

Case report, page 4, line 3: the patient was gasping as a sign of respiratory insufficiency

RESPONSE: The reviewer’s sugegstion was used as follows:- “ ... walking and lost consciousness. Upon arrival of the paramedics, the patient was comatose with a Glasgow Coma Scale of 3 (GCS - E1V1M1) and was gasping as ashowed signs of respiratory insufficiency counting for a massively reduced circulation / hemodynamic arrest through gasping. The peripheral oxygen saturation was 30 % underlining the assessment, the blood pressure was 100/60 mmHg and the patient suffered from a narrow QRS-complex tachycardia with a frequency of 180 bpm with no precise information how long the status persisted. The patient was... “

5. Next, the reviewer asked: In case of collapsing and GCS 3 with gasping it´s a high possibility of massive reduced circulation/hemodynamic arrest. The first measured saturation of 30% is underlining the theory. How long was the time without sufficient circulation? There was no help from bystander?

RESPONSE: The authors seized the idea - the changed text is now: “ ... walking and lost consciousness. Upon arrival of the paramedics, the patient was comatose with a Glasgow Coma Scale of 3 (GCS - E1V1M1) and was gasping as ashowed signs of respiratory insufficiency counting for a massively reduced circulation / hemodynamic arrest through gasping. The peripheral oxygen saturation was 30 % underlining the assessment, the blood pressure was 100/60 mmHg and the patient suffered from a narrow QRS-complex tachycardia with a frequency of 180 bpm with no precise information how long the status persisted. The patient was ... “

6. Following, the reviewer’s question was: Case report :"Initially, the assessment of body core temperature was impossible due to exceeding of the temperature scale but throughout outpatient clinic service, the body core temperature was measured at 42.3°C." What does it mean? Core temperature was initially higher and because of later measurement the true value is missed?

RESPONSE:[missing]

7. The reviewer raised: ECG: cardial axis is unclear, sinus rhythm, we have not! a typical right bundle branch block. It seems to be a brugada type 2 with st-elevation in v1/v2 and typical saddleback morphe. On the ACC 2022 a poster was presented of a hypothermia induced brugada. Maybe was the first rhythm a VF with a spontaneous conversion into sinus rhythm and the liver failure/MODS are a result of ischemia?

RESPONSE: Based on the serious demand by the reviewers to shorten the whole manuscript, the ECG part was substantially shortened - see within the text.

8. The next aspect mentioned by the reviewer was: In my mind, the theory of hemodynamic instability because of tachycardia/ventricular fibrillation and ischemia, which leads to MODS is possible. Maybe the fast rhythm is caused by a hyperthermia induced Brugada-Syndrome. Do you have any information about ecg-dynamics?

RESPONSE: See also above (RESPONSE to point 7), based on the serious demand by the reviewers to shorten the whole manuscript, the ECG part was substantially shortened - see within the text.

However, the authors seized the reviewer’s idea leading to the changes within the text of the “Discussion“ section as follows:

“ ... and resulted in the patient’s death (1). It remains speculative whether MODS/consecutive liver failure are a result of ischemia - in addition, hemodynamic instability because of tachycardia (or even ventricular fibrillation) and ischemia, which lead to MODS (as a theory), is possible. Perhaps, the fast rhythm is caused by a hyperthermia-induced Brugada syndrome (ion channel disease with electrical disturbance of heart function without detectable alteration of the heart tissue [structure], in which life-threatening cardiac irregularities can occur).

In general, heat dissipation ... “

9. The reviewer tried to improve a text passage as follows:

Page 5 , line 3: "In the course of events, myoglobin-level was declining (Fig. 2A) but the leading clinical findings were progressive microcirculatory failure as well as liver and respiratory failure"

Maybe better: Myoglobin-level declined as a result of performing a Myoglobin filter. The clinical situation was worsening with progressive .....

RESPONSE: In addition, the authors seized also this idea suggested by the reviewer:

“ ... (CVVH) with a myoglobin filter. In the course of events, myoglobin-level was declineding as a result of performing a myoglobin filter (Fig. 2A) but the leading clinical ... “

10. The following better English expression was suggested to be substituted:

Page 7 line 13: A limitation of this scientific report is the only case based on which the problem of heat stroke is described.

better: The meaningfulness of this report based on a single case is limited.

RESPONSE: Based on the serious demand by the reviewers to shorten the whole manuscript, the “Limitations“ aspect was completely erased.

11. The reviewer discussed:

The discussion of liver failure and liver transplantation as an ultimate treatment option is very rare. Under the search terms ((heat stroke) AND (liver failure)) AND (liver transplantation) are nearly 40 results, the latest is from 2022.

RESPONSE: As you may see in Table 1, there is a representative case list (“Introduction“ section) summarizing newly reported cases.

12. The reviewer criticized: The references are not current

RESPONSE: Based on the reviewers‘ suggestion, several references were added to the reference list (in particular, see Table 1) - a substantial portion were used to extract single and detailed patient information for a case collection as also recommended - taken together, “the mean age of all references is now much younger“.

Reviewers’ Comments to Revised Submission

Reviewer 1: Paasch, Christoph

Date received: 14-Jul-2023
Reviewer recommendation: **Return to author for minor modifications**
Reviewer overall scoring: **High**

Comments to author:

Congratulation to the manuscript. It is much better now. Nevertheless, the conclusion has not been shortened.

Reviewer 2: Hansen, Michael

Date received: 08-Jul-2023
Reviewer recommendation: **Return to author for minor modifications**
Reviewer overall scoring: **High**

Comments to author:

Thank you for correcting the paper. i´ve only very little modifications. [annotated file provided]

Authors' Response to Reviewer Comments on Revised Submission

Date received: 21-Jul-2023

Response to Reviewer 1:

The reviewer stated:
… "Conclusion" is still not shortened. …

RESPONSE:
We apologize that we overlooked this aspect.
As you can see, the section "Conclusions" of the "Main text" was appropriately shortened as the reviewer suggested

Comments by the Editor-in-Chief Decision on revised version

All reviewer comments were addressed adequately, and the manuscript should be published in its present stage.
